# Refractive error, eye care needs and attitude towards spectacle wearing among older Zanzibari craftswomen and implications for developing women-targeted services: a cross-sectional study

**DOI:** 10.1136/bmjophth-2023-001283

**Published:** 2023-05-29

**Authors:** Ving Fai Chan, Fatma Omar, Adrianna Farmer, Omar Othman, Ai Chee Yong, Christine Graham, Carlos Price-Sanchez, Ronnie Graham, Michelle Fernandes Martins, Eden Mashayo

**Affiliations:** 1 Centre for Public Health, Faculty of Medicine Health and Life Sciences, Queen's University Belfast, Belfast, UK; 2 College of Health Sciences, University of KwaZulu-Natal, Durban, South Africa; 3 Primary Eye Care, Zanzibar Ministry of Health, Zanzibar, Tanzania; 4 Centre for Public Health, Queen's University Belfast, Belfast, UK; 5 Programmes, Vision Aid Overseas, London, UK; 6 Programmes, Vision Care Foundation, Dar-es-Salaam, Tanzania

**Keywords:** epidemiology, public health, vision, optics and refraction

## Abstract

**Background:**

Aged Zanzibari women are in a disadvantaged position, having high demand for near-vision spectacles. Currently, there is no information on the eye health status of craftswomen, which makes planning a women-targeted project to deliver eye health services to older craftswomen in Zanzibar difficult. We assessed the prevalence of vision impairment, refractive error, presbyopia, effective spectacle coverage (distance and near) and attitude towards spectacle wearing among older Zanzibari craftswomen.

**Methods and analysis:**

This was a cross-sectional study. Unaided and presenting distance and near vision of craftswomen 35 years and older were assessed at the women’s co-operatives. We determined the number of those with distance vision poorer than 6/12 and their causes (distance-vision impairment), the number of those with near vision poorer than N8 at 40 cm (presbyopia) and the number of those whose distance and/or near-vision needs were met adequately with their habitual spectacles (effective distance and near spectacle coverages). A piloted and validated questionnaire (15 statements) was used to determine their attitude towards spectacle wearing.

**Results:**

In all, 263 craftswomen participated in the survey (mean age 52.1 years±9.4 years). The prevalence of distance vision impairment among the craftswomen was 29.7% (95% CI 24.2% to 35.6%), the primary cause being uncorrected refractive error (n=51, 65.4%), and none were corrected. The prevalence of presbyopia was 86.6% (95% CI 81.5% to 90.7%, n=231) and the effective near spectacle coverage was 0.99%. The craftswomen showed a positive attitude towards spectacle wearing (strongly agree or agree) based on 12 out of 15 statements.

**Conclusion:**

The high burden of vision impairment, uncorrected distance refractive error and presbyopia, and a positive attitude towards spectacle wearing among older craftswomen in Zanzibar indicated the need for women-targeted eye health programmes in low-resource settings.

**WHAT IS ALREADY KNOWN ON THIS TOPIC**

- Globally, women are disproportionately affected by vision impairment due to poor access to care.
- A previous population-based survey in Zanzibar shows high presbyopia prevalence and low correction rate, but no eye health information among older craftswomen is available.

**WHAT THIS STUDY ADDS**

- Prevalence of vision impairment, distance refractive error and presbyopia among craftswomen is high, but the correction rate is extremely low (closed to 0%).
- Encouragingly, craftswomen had positive attitude towards spectacle wearing.

**HOW THIS STUDY MIGHT AFFECT RESEARCH, PRACTICE OR POLICY**

- A women-targeted eye care programme is needed to address the high burden of eye care needs.

## Introduction

Women and girls are disproportionately affected by the impact of vision impairment and blindness.^1 2^ Globally, they accounted for 56% of the 36 million blind people and 55% of the 217 million people with moderate to severe vision impairment.^3^ As a result, women and girls tend to have less access to education, have decreased employment opportunities, are socially excluded and have a higher risk of experiencing violence compared with their male counterparts.^1^ The key reasons for this inequity include female health not being prioritised, age-related eye diseases linked to higher life expectancy such as presbyopia, age-related macular diseases in women and more limited access to health services.^2^

The immediate eye health needs and the barriers women face call for women-targeted services that directly contribute to their participation in the workforce and productive employment (Sustainable Development Goal (SDG) 8), economic growth, poverty alleviation (SDG 1) and gender equity (SDG 5). In particular, a decline in unaided near vision due to ageing or presbyopia is prevalent during the working years.^4^ Globally, presbyopia is the most common cause of vision impairment, affecting more than 1 billion people.^5^ However, a recent review^6^ has shown that several studies^7–9^ found that near-vision spectacle provision can be a low-cost, sustainable and effective approach to improving work productivity. The PROductivity Study of Presbyopia Elimination in Rural-dwellers (PROSPER) trial^7^ among tea pickers in India, of which 78% were women, showed a significant increase in work productivity with an effect size of 1.01 (95% CI 0.86 to 1.16, p<0.0001) with presbyopic spectacle correction. The same was observed in Naidoo *et al*’s^8^ and Chan *et al*’s^9^ prospective studies among Durban textile workers who were predominantly women (>90%), where work productivity (improved by 6.4%, 95% CI 5.2% to 7.7%) and quality of life scores (improved by 21.9, 95% CI 16.7 to 27) increased significantly after correction. As presbyopia typically occurs during active working years; uncorrected presbyopia causes a great economic burden estimated at US$25 billion in global productivity loss in 2018.^4^

Furthermore, we reviewed the literature from the last decade and identified only three eye health programmes dedicated to women in low-income and middle-income countries: the Improving Vision to Empower Female Factory Workers programme in Vietnam,^10^ the See to Earn programme in Kenya^11^ and a collaborative programme in Bangladesh^12^ that aimed to empower women by providing eye health services at the workplace. At the end of the 2-year Improving Vision to Empower Female Factory Workers programme in Vietnam, the female workers were found to have increased knowledge in eye health and better eye practice and felt more confident at work due to better product quality and productivity (from 87.6% to 91.2%).^10^ An analysis of the eye health programme data in Bangladesh also found that female garment workers with near-vision impairment earned $13.3 less per month than those without vision impairment and argued that correction might be able to address gender inequity issues in the workplace.^12^

This gap in women-targeted eye health services led to the conception of the Women’s Empowerment through Investing in Zanzibari Craftswomen’s Eyesight (WE-ZACE)^13^ cohort study that aims to determine the level of empowerment among craftswomen by correcting their near-vision impairment (presbyopia) using spectacles. The official statistics in Zanzibar^14^ show that in 2019, 23% of Zanzibari women headed a household (ie, primary person to provide for the household); each Zanzibari woman head supported an average of nine unemployed persons; and 44% of the women had no education. Woman heads play multiple roles such as working in informal and low-income jobs while also being responsible for household chores. Many women generate income by working as craftswomen^14^ in women’s co-operatives, which involves activities heavily dependent on good near vision, such as weaving, sewing and pottery. However, a population-based survey in 2010 showed that the prevalence of presbyopia among people 40 years and older in Zanzibar was 89.2%, but the correction rate was a mere 17.6%.^15^ Early engagement with the co-operatives indicated that most women entrepreneurs were 35 years and older, presbyopic and yet to be corrected.^13^

Consumer attitudes towards a health product might play a role in the uptake of the product, in this case, spectacles. Although studies have been conducted to understand attitudes towards refractive error and spectacle wearing among children, only one^16^ was conducted to explore this issue among adult and older adult populations. A community survey in Northwest Ethiopia found that among 780 adult populations, 90.4% had a favourable attitude towards spectacle use.^16^ Due to this paucity of information, we consulted with local stakeholders before starting the WE-ZACE project. We identified a great need for spectacles and addressing eye irritation at the workplace among craftswomen, and the main barrier to eye health is poor attitude towards eye health.^13^

The disadvantaged position of women, the high demand for near-vision spectacles and low spectacle coverage in Zanzibar are reasons for WE-ZACE deciding to work among older craftswomen. Culturally, the Zanzibari population is predominantly Muslim with a deep-rooted patriarchal society, where gender roles are well defined (ie, men are providers for women; women should be submissive and obey their husbands). We understand that information on the eye health status of craftswomen and their attitudes towards wearing spectacles will be critical in planning a women-targeted, need-specific and culturally sensitive project to deliver eye health services to older craftswomen in Zanzibar. Hence, this study aims to fill this information gap.

## Materials and methods

The study was a cross-sectional analysis conducted as part of the bigger WE-ZACE study on both Unguja and Pemba, the two main islands of Zanzibar, from 4 April to 21 April 2022.

### Patient and public involvement (PPI)

Community beneficiaries (craftswomen, co-operative managers and leaders) were involved in the development of the WE-ZACE programme through four PPI meetings during the initiation and development stage. They provided input on the research questions, suggestions on timing for recruitment and implementation.

### Participants

We included all registered women co-operatives (The Ministry of Labour, Youth, Women and Children Development Zanzibar, F.Omar, personal communication, 2021) that hosted craftswomen in the study (10 from Unguja Island and 9 from Pemba Island). We invited all craftswomen who were 35 years old and older to participate in the survey (finite sampling). Women who were not involved in craftwork (eg, farming and selling products) were excluded. There were 297 craftswomen (173 in Unguja and 124 in Pemba) registered at these women co-operatives.

### Data collection

There were four teams of data collectors, each consisting of two ophthalmic clinical officers who conducted eye health screenings and examinations and one survey administrator. The four teams underwent a 5-day training to standardise the survey procedures and data recording. Prior to the implementation of the survey, written informed consent was sought from all craftswomen participating in the study. The survey was supervised by three research team leaders (FO, EM and OO). Eye examinations began with assessment of the unaided and presenting distance vision (monocularly) under normal lighting using a modified Snellen Tumbling E-Chart (a chart routinely used in Zanzibar’s adult vision screening protocol). If the craftswomen were current spectacle wearers, their aided presenting vision would be tested. Those who could not identify at least four out of five letters on the 6/12 line in the better eye were considered to have failed vision screening and were categorised into the ‘distance-vision impairment <6/12’ group. Craftswomen were categorised as ‘having normal vision ≥6/12’ if they could read four out of five letters on the 6/12 line or better in the better eye and if they wore spectacles. Further eye health examination using a torch and direct ophthalmoscope under dim lighting was conducted to determine the cause of vision impairment. Subjective refraction for craftswomen with uncorrected refractive error was conducted on-site to determine the spectacle prescriptions. Those who passed distance-vision screening and those with vision impairment due to uncorrected refractive error but correctable to better than 6/12 were then tested for their near vision at 40 cm and their usual working distance. The craftswomen were considered to fail near-vision screening if they could not read at N8 at 40 cm binocularly. Further testing was done to determine if their near vision could be corrected with near spectacle correction. Near spectacle correction (ready-made or custom-made) was prescribed at their usual working distance. Those who had eye conditions which needed further ophthalmological assessment were referred to the nearest eye clinic for management.

Subsequently, the data collectors administered a piloted and validated survey questionnaire to collect demographic information, which included age (later grouped into 35–44, 45–55 and older than 55 years old); residence locations (Unguja or Pemba); marital status (married, single or widowed); type of craftwork engaged; years working as craftswomen; the number of children and dependents (grouped into intervals of 4); and monthly income. Ocular and spectacle-wearing history, eye health and information about accessing spectacles were also determined. The questionnaire ended with 15 statements exploring the craftswomen’s attitudes towards spectacle wear, which were individually rated on a 5-point Likert scale (5=strongly agree, 4=agree, 3=neutral, 2=disagree and 1=strongly disagree). These 15 statements were decided after discussions with local stakeholders.

### Data analysis

The Statistical Package for Social Science V.25.0 was used for data management and analysis. The data were cleaned, checked for inconsistencies by OO and VFC, and analysed by AF, ACY and VFC. The main outcomes to determine the craftswomen’s eye health needs were guided by the WE-ZACE Theory of Change^13^ and discussion with the Ministry of Health, Social Welfare, Empowerment, Gender and Children and Tanzanian Optometry Association. These primary outcomes included the prevalence of distance-vision impairment, presbyopia, and effective distance and near spectacle coverage. Secondary outcomes were ocular and spectacle history and attitude towards spectacles. The prevalence of distance-vision impairment was defined using presenting distance vision worse than 6/12 in the better eye. If their vision could be improved to better than 6/12 with spectacles, they were classified as having an uncorrected refractive error. The magnitude of refractive error was presented in terms of spherical equivalent, which was the sum of the spherical power and half of the cylindrical power. Presbyopia was defined using presenting near vision failing N8 at 40 cm but correctable to better than N8 at 40 cm with spectacles. Effective spectacle coverage for both distance and near was calculated as follows:

Effective refractive error coverage (distance refractive error)=[number of craftswomen with distance unaided VA <6/12 but corrected to VA ≥6/12 with current habitual distance spectacles/total number of craftswomen with distance refractive error]×100%.

Effective refractive error coverage (presbyopia)=[number of craftswomen with near unaided VA <N8 at 40 cm but corrected to near VA ≥N8 at 40 cm with current habitual near spectacles/total number of craftswomen with presbyopia]×100%

Logistic regressions were used to investigate the association of demographic profiles with the main outcomes with ORs reported. CIs and p values (significant at the p<0.05 level) were calculated. Descriptive statistics on the responses to attitude statements are presented in percentages. We reported our findings per the the Strengthening the Reporting of Observational Studies in Epidemiology Statement: Guidelines for Reporting Observational Studies.

## Results

A total of 282 potential women were identified by the women’s co-operative managers and invited to participate in the study from 7 to 24 April 2022. Four were excluded as they were younger than 35 years old, and a further 15 were excluded as they were farmers. The final sample size was 263 craftswomen, with a coverage of 88.6% (85.5% in Unguja and 92.7% in Pemba). The mean age of craftswomen was 52.1 years (SD 9.4), with the highest proportion of craftswomen being in the age cohort of 45–55 years (n=110, 41.8%), followed by those older than 55 years (n=86, 32.7%). Regarding duration engaged in their craftwork, the majority were weavers (n=149, 56.7%) who had been involved in their craftwork for 10–20 years (n=165, 62.7%). In terms of their social indicators, most were married (n=192, 73.0%), had five to eight children (n=138, 53.7%), were supporting one to five dependents (n=139, 52.9%) and earned less than TSH100 000 (US$42.90) a month (n=232, 88.2%). The median monthly income was TSH40 000 (IQR TSH25 000–TSH60 000) (equivalent to US$17.15, IQR US$10.72–US$25.73) (table 1).

**Table 1:** Demographic profiles of Zanzibari craftswomen (n=263)

	n	Percentage (%)
Locations		
Unguja	148	56.30
Pemba	115	43.70
Age group (years)		
35–44	67	25.50
45–55	110	41.80
Older than 55	86	32.70
Mean age: 52.1±9.4		
Level of education		
No formal education	41	15.70
Did not complete primary education	37	14.20
Completed primary education	114	43.70
Completed secondary education	69	26.40
Missing data=2		
Type of craftwork engaged		
Weaving	149	56.70
Tailoring and sewing	80	30.40
Pottery	10	3.80
Producing oil and making soaps	24	9.10
Years working as craftswomen		
Less than a year to 10 years	0	0
>10–20 years	165	62.70
>20–30 years	79	30.00
>30–40 years	13	4.90
Missing data=1	5	1.90
Marital status		
Single	3	1.10
Married	192	73.00
Widowed	41	15.60
Separated/divorced	27	10.30
Number of children		
1–4	76	29.60
5–8	138	53.70
More than 8	43	16.70
Missing data=6		
Number of dependents		
None	26	9.90
1–4	139	52.90
5–8	85	32.30
More than 8	13	4.90
Personal monthly income level		
No income	4	1.50
Less than TSH100 000 (US$42.90)	232	88.20
More than TSH100 000 (US$42.90) to TSH200 000 (US$84.80)	20	7.60%
More than TSH200 000	7	2.70
Median: TSH40 000 (IQR TSH25 000–TSH60 000) or US$17.15 (IQR US$10.72–US$25.73)		

The prevalence of distance-vision impairment among the craftswomen was 29.7% (95% CI 24.2% to 35.6%). The primary cause of distance-vision impairment was uncorrected refractive error (n=51, 65.4%) followed by cataract (n=18, 23.1%). Among those with uncorrected refractive error (n=51), 10 were current spectacle wearers, but none were corrected to 6/12 or better, making the effective spectacle coverage 0%. Most had low myopia (−0.50 to −3.00 D; n=26, 51.0%) and low hyperopia (+0.50 to +1.75 D; n=16, 31.4%).

Two hundred and thirty-one (231) craftswomen had presbyopia, indicating a prevalence of 86.6% (95% CI 81.5% to 90.7%) in the cohort. Seven craftswomen (3.03%) were current near spectacle wearers, but only two were adequately corrected with habitual correction, making the effective near spectacle coverage 0.99%. Of the craftswomen diagnosed with presbyopia and provided with spectacles, most were corrected with an addition of +2.00 D to +2.75 D (n=93, 47.2%).

About two-thirds of the craftswomen previously had an eye examination (n=165, 62.7%), with most (n=105, 63.6%) being conducted within the last 2 years. Most of these previously examined craftswomen also reported that they would go to a public hospital if they needed a pair of spectacles (n=133, 80.5%). Many could access eyeglasses services nearby within the village (n=120, 45.6%), but about a quarter (n=60, 22.7%) did not know where to get their eyeglasses (table 2).

**Table 2:** Eye health status among craftswomen

	Number	Percentage (%) (95% CI)
Distance vision		
Distance presenting vision in the better eye (n=263)		
Normal vision	185	70.3 (64.4–75.8)
Vision impairment	78	29.7 (24.2–35.6)
Refractive error	*51*	65.4 (53.8–75.8%)
Cataract	*18*	23.1 (14.3–34.0)
Maculopathy	2	2.56 (3.00–9.00)
Retinal disorders	1	1.28 (0.00–6.90)
Unknown causes	6	7.69 (2.90–16.0)
Current distance spectacle wearer out of those with refractive error (n=51)		
Yes, and presenting vision was 6/12 or better*	0	0.00
Yes, but presenting vision was worse than 6/12	10	19.6 (8.71–30.5)
Not a current distance spectacle wearer	41	80.4 (69.5–91.3)
Magnitude of distance refractive error (n=51)		
+2.00 D to +5.00 D	4	7.84 (2.20–18.9)
+0.50 D to +1.75 D	16	31.4 (19.1–45.9)
+0.25 D to −0.25 D	3	5.88 (1.20–16.2)
−0.50 D to −3.00 D	26	51.0 (36.6–65.2)
−3.25 D to −6.00 D	1	1.96 (0.00–10.4)
Missing data=1*		
Near vision		
Near-vision testing† (n=231)		
Pass N8 at 40 cm	31	13.4 (9.30–18.5)
Fail N8 at 40 cm	200	86.6 (81.5–90.7)
Current near spectacle wearer out of those with presbyopia (n=202)		
Yes, and near presenting vision N8 or better‡	2	0.99 (0.00–2.36)
Yes, but near presenting vision worse than N8	5	2.48 (0.333–4.62)
Not current near spectacle wearer	195	96.5 (94.0–99.1)
Prescription of near reading glasses (n=200)		
+1.00 D to +1.75 D	57	28.9 (22.5–34.9)
+2.00 D to +2.75 D	93	47.2 (41.1–54.8)
>+2.75 D	47	23.9 (18.2–30.0)
Missing data=3*		
Prior eye examination (n=263)		
Yes	165	62.7 (56.6–68.6)
Less than a year ago	38	23.0 (16.8–30.2)
1–2 years ago	67	40.6 (33.0–48.5)
More than 2 years ago	60	36.4 (29.0–44.2)
No	98	37.3 (31.4–43.4)
Where to access spectacle (n=165)		
Government hospital	133	80.5 (73.6–86.3)
Outreach	27	16.0 (11.1–23.0)
Private/charitable hospital	5	3.05 (1.00–7.00)
Location to get eyeglasses		
Nearby in the neighbourhood in the same village	120	45.6% (39.6–51.6)
At a moderate distance from the neighbourhood in the same village	14	5.30 (2.61–8.04)
Different villages within the same district	4	1.50 (0.20–2.84)
Far from the neighbourhood in the same village	32	12.2 (2.57–16.6)
Different district	33	12.5 (8.54–16.6)
I do not know where to get eyeglasses	60	22.8 (17.7–27.9)

*Effective distance spectacle coverage.

†Prevalence of presbyopia.

‡Effective near spectacle coverage.

Multivariate analysis showed that those older than 55 years old were 5.27 times (95% CI 1.78 to 15.6) more likely to have a distance-vision impairment than those 35–45 years old, and those who had completed primary education were 0.37 times (95% CI 0.15 to 0.95) less likely to have a vision impairment than those with no education. Those with one to four and five to eight dependents were 4.77 times (95% CI 1.24 to 18.4) and 6.35 times (95% CI 1.10 to 36.6) more likely to have uncorrected presbyopia than those with no dependents, respectively (table 3)

**Table 3:** Association between vision impairment (n=78) and uncorrected presbyopia (n=200) and the craftswomen’ demographic profiles (multivariate analysis)

	Vision impairment OR (95% CI) N=78	Presbyopia OR (95% CI) N=200
Age group (years)		
35–44	Reference	Reference
45–55	2.23 (0.85–5.82)	2.83 (0.92–8.64)
Older than 55	5.27 (1.78–15.6)	1.33 (0.34–5.16)
Level of education		
No formal education	Reference	Reference
Did not complete primary education	0.63 (2.14–1.88)	1.43 (0.31–6.51)
Completed primary education	0.37 (0.15–0.95)	2.71 (0.71–10.4)
Completed secondary education	0.94 (0.36–2.47)	4.30 (0.87–21.2)
Type of craftwork engaged		
Weaving	Reference	Reference
Tailoring and sewing	0.53 (0.24–1.16)	1.16 (0.40–3.32)
Pottery	1.03 (0.23–4.57)	–
Producing oil and making soaps	0.57 (0.17–1.92)	1.02 (0.22–4.66)
Years working as craftswomen		
1–10	Reference	Reference
>10–20	1.70 (0.86–3.38)	0.63 (0.24–1.65)
>20–30	1.27 (0.35–4.62)	–
>30–40	0.72 (0.05–9.71)	–
Marital status		
Single	Reference	–
Married	0.67 (0.04–13.1)	Reference
Widowed	1.14 (0.05–24.5)	0.97 (0.24–3.85)
Separated/divorced	1.01 (0.05–22.3)	4.18 (0.64–27.2)
Number of children		
1–4	Reference	Reference
5–8	1.05 (0.49–2.24)	1.16 (0.39–3.47)
More than 8	0.51 (0.16–1.70)	–
Number of dependents		
None	Reference	Reference
1–4	1.59 (0.51–4.99)	4.77 (1.24–18.4)
5–8	2.27 (0.60–8.65)	6.35 (1.10–36.6)
More than 8	2.09 (0.22–19.9)	–
Personal monthly income level		
Less than TSH100 000	Reference	Reference
TSH100 000–TSH200 000	1.44 (0.42–4.89)	0.49 (0.11–2.21)
More than TSH200 000	4.27 (0.75–24.4)	0.65 (0.06–7.46)

Overall, the craftswomen showed a positive attitude towards spectacle wearing to 12 (80%) of the 15 statements (more than 75% disagreed or strongly disagreed with the statements), while responses to only three statements (20%) did not. Responses to these three statements yielded a negative attitude towards spectacle wearing from the following results: (1) 98 craftswomen (37.3%) felt that people wearing spectacles look more educated; (2) 68 craftswomen (25.9%) felt that people wearing spectacles look fashionable; and (3) 51 craftswomen (19.3%) believed people wearing spectacles are bringing attention to themselves to show off (table 4).

**Table 4:** Craftswomen’s attitudes towards spectacle wear (N=263)

Statements	Strongly agree, n (%)	Agree, n (%)	Neutral, n (%)	Disagree, n (%)	Strongly disagree, n (%)
Wearing spectacles does not help correct vision.	3 (1.11)	6 (2.30)	8 (3.00)	152 (57.8)	94 (35.7)
Wearing spectacles will make eye problems worse.	5 (1.90)	12 (4.60)	28 (10.6)	137 (52.1)	81 (30.8)
Wearing spectacles will cause more headaches or tearing.	5 (1.90)	20 (7.60)	33 (12.5)	126 (47.9)	79 (30.0)
People wearing spectacles look more educated.	17 (6.50)	81 (30.8)	23 (8.70)	83 (31.6)	59 (22.4)
People wearing spectacles look fashionable.	12 (4.60)	56 (21.3)	14 (5.30)	116 (44.1)	65 (24.7)
People wearing spectacles have more money or are from higher social status.	5 (1.90)	22 (8.40)	15 (5.70)	134 (51.0)	87 (33.1)
People wearing spectacles look older.	9 (3.40)	16 (6.10)	1 (0.400)	140 (53.2)	97 (36.9)
People wearing spectacles look disabled.	8 (3.00)	24 (9.10)	7 (2.70)	140 (53.2)	84 (31.9)
People wearing spectacles are bringing attention to themselves to show off.	13 (4.90)	38 (14.4)	18 (6.80)	117 (44.5)	77 (29.3)
Wearing spectacles harms a woman’s prospects of marriage.	6 (2.30)	8 (3.00)	13 (4.90)	132 (50.2)	104 (39.5)
Wearing spectacles would make me less likely to choose someone as a marriage partner.	2 (0.80)	10 (3.80)	10 (3.80)	148 (56.3)	93 (35.4)
If I wear spectacles, people will think better of me as a craftswoman.	9 (3.40)	22 (8.40)	12 (4.60)	138 (52.5)	82 (31.2)
If I wear spectacles, people will be less likely to order products from me.	5 (1.90)	13 (4.90)	11 (4.20)	145 (55.1)	89 (33.8)
If I wear spectacles, people will assign me to less technical or complicated work.	9 (3.40)	21 (8.00)	19 (7.20)	136 (51.7)	78 (29.7)
If I wear spectacles, people will bully me or tease me.	12 (4.60)	30 (11.4)	12 (4.60)	116 (44.1)	93 (35.4)

## Discussion

The study aimed to understand the eye health needs among older Zanzibari craftswomen and found that the prevalence of vision impairment and presbyopia among this population was high (about 30% and 87%, respectively), with extremely low spectacle correction rates (0% and 0.99%, respectively). Uncorrected refractive errors remained the primary cause of vision impairment, and the overall attitude towards spectacle wearing was positive. These findings will be useful for planning the WE-ZACE programme.

About 3 in 10 craftswomen had distance-vision impairment, the two main causes being uncorrected refractive error (65.4%) and cataract (23.1%). More concerningly, none of them was corrected. Not only have we found a significant spectacle and eye health need among the craftswomen, but also under the supposition that non-eye care personnel are used for eye health screening, adequate training will be required to detect these two conditions and the existence of a continuum of care for these people.

Our cohort’s prevalence of presbyopia (86.6%) was similar to population-based presbyopia surveys in Zanzibar (89.2%)^15^ and Kenya (85.4%)^17^ but much higher than that of Eritrea (32.9%),^18^ Uganda (50.3%)^19^ and Tanzania (61.7%^20^ and 46.5%^21^). These variances in prevalence might be due to differences in the cohort’s age, survey populations, presbyopia definitions and survey designs. Among participants in our cohort, only two people were corrected adequately with their current spectacles. Laviers *et al*’s survey observed much higher coverage (17.6%),^15^ but most other surveys also reported very low coverage (0% in Uganda,^19^ 0.42% in Tanzania,^21^ 6.3% in Kenya^17^ and 9.9% in Eritrea^18^). Our findings suggested that being older, having more dependents and the long travelling distance to the eye centre may be barriers to correction (table 3). Furthermore, official statistics showed that Zanzibari women were two times more likely to be uneducated and three times more likely to be unemployed than men.^14^ If the women were employed, 73% were paid less than their husbands.^14^ With the majority in our cohort earning less than TSH100 000 (US$42.90) per month, we hypothesised that our craftswomen might have cost and priority issues, which were also reported among older adults in Kenya,^17^ Eritrea,^18^ Uganda^19^ and Tanzania.^21^

Two eye care facilities in Zanzibar currently provide refractive services (one on each island). These facilities are located in well-populated areas. While active health-seeking behaviour is encouraged, our findings may indicate that WE-ZACE is important in addressing the immediate refractive error and presbyopia needs among older craftswomen by bringing services closer to them at this early implementation stage of the WE-ZACE programme. Many women in Zanzibar have been deprived of basic needs and opportunities due to gender-specific barriers and restrictions, such as perceiving women’s role in society as submissive. Women are also expected to be obedient daughters, wives and mothers and hence do not have the autonomy to make decisions for themselves. These restrictions might have caused women to have less education and financial freedom than men and reduced healthcare access. Addressing eye health needs may also improve women’s ability to work more efficiently, generate greater income and achieve financial freedom, which is closely aligned with the Zanzibar Strategy for Growth and Reduction of Poverty.^22^ An advantage of working with the women co-operatives is that they are organised in groups that can be more efficiently contacted, thereby reducing associated programme costs. Furthermore, with a bigger group of women entrepreneurs in Zanzibar beyond the WE-ZACE programme yet to be screened, we foresee a greater need and, subsequently, a greater impact for such a women-targeted programme.

More than half of our cohort (54.4%) were required to travel a significant distance to purchase spectacles or did not know where to get their eyeglasses. Combined with the fact that 76.1% (n=150) of craftswomen with final prescriptions (+1.00D to +2.75 D) (table 2) were able to be corrected with ready-made near-vision spectacles through this study, WE-ZACE and future programmes could benefit from providing ready-made spectacles to reduce the cost to both the programme and the women. Not only is a pair of ready-made spectacles three times cheaper than custom-made spectacles in Zanzibar (US$5 for ready-made spectacles vs US$15 for custom-made spectacles) but also the on-site prescribing will reduce the loss of follow-up and logistic costs associated with multiple visits to the eye centre for eye examination and prescription. According to the spherical equivalent power of the cohort, another 88.3% of their distance-vision spectacles magnitude fell under the ready-made spectacle inventories and could potentially be corrected with ready-made spectacles. However, in the long run, craftswomen should be sensitised and enabled to seek treatment voluntarily, given that the Zanzibar National Health Scheme, which covers the costs of eye health services in government facilities, will be launched in September 2022.

Our findings, which showed that craftswomen had a positive attitude towards spectacle wearing, were similar to findings from Northwest Ethiopia^16^ and Nigeria.^23^ These attitude findings can help develop an effective WE-ZACE strategy and create persuasive awareness campaigns. We believe that a positive attitude towards spectacles among a population with such a low correction rate is critical because attitude plays a central role in accepting a relatively new intervention and significantly influences the success of the WE-ZACE strategy. Furthermore, with a pair of near-vision spectacles costing an average of US$5–US$7 in Zanzibar, they might be marginally affordable to the craftswomen as majority of women earn less than US$43 per month (table 1). Apart from using the near-vision spectacles for work, craftswomen may also use them for reading Qu’ran, in performing housework such as sorting stones from rice and cutting vegetables, activities most homemakers in Zanzibar engaged in. We hypothesise that satisfaction with visual needs and a positive attitude will translate into improved wearing adherence and future procurement.

There are a few limitations to be acknowledged. First, we included only officially registered women’s co-operatives, and personal communications with local stakeholders revealed that several co-operatives operate independently, unregistered. This may have affected our findings. Second, we have included only older craftswomen (35 years and older) and may have overlooked the eye conditions of craftswomen younger than our cohort. Third, we have included only women involved in craftwork and did not include those involved in non-craftwork (agriculture and seaweed farming) and hence did not estimate the needs of other women entrepreneurs working at the women’s co-operatives.

## Conclusion

Our study highlighted the high burden of vision impairment, uncorrected refractive error and presbyopia and a positive attitude towards spectacle wearing among older craftswomen in Zanzibar. The findings have three main implications. First, we have brought the craftswomen’s eye health issues to the forefront with gender-specific statistics, thereby highlighting one of the most persistent global gaps in spectacle coverage. Our findings will contribute to the Zanzibar Inclusive Data Charter Action Plan^24^ on ‘enhancing the production, availability, and use of gender statistics’. Second, the data could help in programme planning and could be used to advocate for initiatives to enable women to be financially and socially independent. Third, the potential of good spectacle-wearing acceptance and adherence may be achieved. Through women-specific data and women-targeted health programmes like WE-ZACE, we could address gender inequality issues in Zanzibar and low-income and middle-income countries through improved eye health.

## Data Availability

Data are available in a public, open access repository. Dataset could be accessed from QUB PURE repository (https://doi.org/10.17034/e4e5125f-8cd9-41cd-8af1-ab317ab07fd7).
